# Low-dose *in situ* prelocation of protein microcrystals by 2D X-ray phase-contrast imaging for serial crystallography

**DOI:** 10.1107/S2052252520013238

**Published:** 2020-10-23

**Authors:** Isabelle Martiel, Chia-Ying Huang, Pablo Villanueva-Perez, Ezequiel Panepucci, Shibom Basu, Martin Caffrey, Bill Pedrini, Oliver Bunk, Marco Stampanoni, Meitian Wang

**Affiliations:** a Paul Scherrer Institute, Forschungsstrasse 111, Villigen, 5232, Switzerland; b Synchrotron Radiation Research and NanoLund, Lund University, Box 118, Lund, 221 00, Sweden; c EMBL Grenoble, 71 avenue des Martyrs, Grenoble, 38042, France; d School of Medicine and School of Biochemistry and Immunology, Trinity College, Dublin 2, D02 R590, Ireland; e Institute of Biomedical Engineering, University and ETH Zurich, Zurich, 8092, Switzerland

**Keywords:** serial crystallography, X-ray imaging, prelocation, automated data collection, structural biology, membrane proteins, macromolecular crystallography beamlines, flat geometry

## Abstract

Serial protein crystallography has emerged as a powerful method of data collection on small crystals from challenging targets, such as membrane proteins. Multiple microcrystals need to be located on large and often flat mounts while exposing them to an X-ray dose that is as low as possible. A crystal-prelocation method is demonstrated here using low-dose 2D full-field propagation-based X-ray phase-contrast imaging at the X-ray imaging beamline TOMCAT at the Swiss Light Source (SLS). This imaging step provides microcrystal coordinates for automated serial data collection at a microfocus macromolecular crystallography beamline on samples with an essentially flat geometry. This prelocation method was applied to microcrystals of a soluble protein and a membrane protein, grown in a commonly used double-sandwich *in situ* crystallization plate. The inner sandwiches of thin plastic film enclosing the microcrystals in lipid cubic phase were flash cooled and imaged at TOMCAT. Based on the obtained crystal coordinates, both still and rotation wedge serial data were collected automatically at the SLS PXI beamline, yielding in both cases a high indexing rate. This workflow can be easily implemented at many synchrotron facilities using existing equipment, or potentially integrated as an online technique in the next-generation macromolecular crystallography beamline, and thus benefit a number of dose-sensitive challenging protein targets.

## Abstract 2

A microcrystal-prelocation method is demonstrated using low-dose 2D full-field propagation-based X-ray phase-contrast imaging on samples with an essentially flat geometry for automated serial crystallography data collection at a microfocus macromolecular crystallography beamline.

## 1. Introduction   

Macromolecular crystallography (MX) data collection at X-ray free-electron lasers (XFELs) requires the merging of still images from many crystals exposed to intense femto­second X-ray pulses, hence its commonly given name of serial femtosecond crystallography (SFX) (Chapman *et al.*, 2011 ▸; White *et al.*, 2012 ▸, 2016 ▸). The development of SFX has sparked a renewed interest in multi-crystal methods at storage-ring-based synchrotron radiation sources (‘synchrotrons’ in the following), with the additional possibility of collecting images as series of sample rotations of individual crystals covering a wedge in angular space (‘wedges’ in the following), instead of pure stills (Gati *et al.*, 2014 ▸; Huang *et al.*, 2015 ▸; Zarrine-Afsar *et al.*, 2012 ▸). The term serial crystallography has been retained for this synchrotron data-collection protocol and underlines the common ground with SFX, although the series collected differ from SFX by the relation between consecutive images and the rotation during each frame. Concurrently, various sample-delivery methods specifically suited for serial crystallography have been developed for synchrotron use in a high-throughput and/or routine manner. They can be roughly classified into two groups – injection methods (Botha *et al.*, 2015 ▸; Cheng, 2020 ▸; Martin-Garcia *et al.*, 2017 ▸; Nogly *et al.*, 2015 ▸; Stellato *et al.*, 2014 ▸) and fixed-target methods (Coquelle *et al.*, 2015 ▸; Huang *et al.*, 2015 ▸; Zarrine-Afsar *et al.*, 2012 ▸). Among the fixed-target approaches, the double-sandwich *in situ* method (Axford *et al.*, 2016 ▸; Broecker *et al.*, 2016 ▸; Huang *et al.*, 2015 ▸, 2016 ▸, 2018 ▸) was used both at room temperature or in cryogenic conditions. This method is characterized by a flat geometry, since the sample was grown between two thin and flat films. This specific geometry enables data collection on hundreds of crystals spread on its large area with small wedges. The maximum rotation angle is in practice limited by the tolerable X-ray dose and by the geometry. Lipid cubic phase (LCP) is often used as a growth medium (Axford *et al.*, 2015 ▸; Broecker *et al.*, 2016 ▸; Huang *et al.*, 2015 ▸, 2016 ▸, 2018 ▸). The double-sandwich *in situ* method is of particular relevance for small dose-sensitive crystals, which are therefore hardly amenable to harvesting and single-crystal data collection, *e.g.* membrane-protein crystals. Serial methods make it possible to expose these small crystals to their maximum safely tolerable dose over a small rotation range, thereby maximizing resolution, completeness and multiplicity by merging data from a number of crystals.

Collecting wedges first requires finding and centering the crystals in the X-ray beam. At XFELs, stills data collection does not strictly require centering but prelocation methods are of interest to increase the hit rate and sample efficiency, *i.e.* the number of useful images collected for a given sample, or ultimately the amount of protein required to collect a full dataset (Martiel *et al.*, 2019 ▸; Oghbaey *et al.*, 2016 ▸). Key properties to be considered for crystal location are the dose applied to the crystals, specificity to protein and/or crystalline material, accuracy of the determined crystal coordinates, time efficiency (*i.e.* beam time needed and other offline resources), and the effective availability of the technique at beamlines. The crystal-locating step can be performed online, *i.e.* directly on the mounted sample immediately before data collection. The majority of synchrotron microfocus beamlines offer workflows and pipelines specifically tailored to perform serial crystallography on goniometer-mounted samples in an automatic or semi-automatic manner. This is based on a raster (or grid scan) to locate crystals in a potentially opaque sample from their diffraction signal, followed by automated wedge data collection, and assisted processing and merging (Aishima *et al.*, 2010 ▸; Basu *et al.*, 2019 ▸; Bowler *et al.*, 2010 ▸; Cherezov *et al.*, 2009 ▸; Guo *et al.*, 2018 ▸; Hirata *et al.*, 2019 ▸; Melnikov *et al.*, 2018 ▸; Wojdyla *et al.*, 2016 ▸; Zander *et al.*, 2015 ▸). Raster location provides a diffraction-specific signal; however, a potentially significant fraction of the tolerable dose is applied to the crystals, particularly for weakly diffracting ones. The time efficiency and accuracy in crystal location depend on many beamline parameters (beam size, flux, detector frame rate, goniometer precision and speed, *etc.*).

Alternative online crystal-location methods by X-ray imaging have been reported. However, these methods require specific equipment and beamline construction or configurations to be performed online, and are applicable only at a limited number of MX beamlines (Polikarpov *et al.*, 2019 ▸; Wojdyla *et al.*, 2016 ▸). Following the pioneering work by Brockhauser *et al.* (2008 ▸) at the ID14-4 MX beamline of the European Synchrotron Radiation Facility and Warren *et al.* (2013 ▸) at the I04 and I03 beamlines at Diamond Light Source, Polikarpov *et al.* (2019 ▸) recently used the homogeneous area of the unfocused beam at the P14 MX beamline at DESY PETRA III to perform full-field X-ray imaging. Two-dimensional projections were recorded and assembled via computed tomography into 3D tomograms of a 3D sample down to micrometre resolution. The configuration change between imaging and data-collection modes was reported to take ∼30 s and the tomographic imaging and reconstruction took ∼1.5 min, for a dose of ∼15 kGy, which is a small fraction of the reported dose in a raster location of 560 kGy. Crystal location is based on the identification of solid objects (segmentation) in the 3D volume and therefore requires acquisition of a full 3D tomogram. 2D X-ray microscopy was also reported at sub-micrometre resolution, but for a dose comparable with raster location. Scanning transmission X-ray microscopy using an attenuated top-hat microbeam has been reported for low-dose online detection of microcrystals at the X06SA MX beamline of the Swiss Light Source (SLS) (Wojdyla *et al.*, 2016 ▸). The X-ray imaging techniques are generally low dose but have the drawback of not being crystal- or protein-specific. Kissick *et al.* (Kissick *et al.*, 2013 ▸; Madden *et al.*, 2013 ▸) used a more crystal-specific imaging method, second-harmonic generation (SHG) from a UV–Vis excitation, also called second-order nonlinear optical imaging of chiral crystals, to locate chiral microcrystals at the 23-ID-B (GM/CA) beamline of the Advanced Photon Source (APS). The setup was then extended to multimodal imaging with two-photon-excitation ultraviolet fluorescence (Newman *et al.*, 2016 ▸). These optical online techniques apply no X-ray dose to the sample, although UV-induced damage can exist. They are, in principle, crystal specific with the exception of non-chiral space groups, and work even with very small crystals. However, they are expensive, which limits their laboratory use, and beamlines offering online SHG are very rare owing to the challenging hardware requirements.

Offline prelocation methods are aimed at locating the crystals on a different instrument and therefore require fiducials as a reference on the sample to register and transfer the coordinates of the crystals on the sample. This reference must be detectable both with the prelocation instrument and at the beamline where the serial data collection is finally performed. The coordinate transfer represents an additional potential factor of precision loss compared with direct online methods. Barnes *et al.* (2019 ▸) reported a UV-based offline prelocation method where coordinate referencing is carried out by clicking the corners of the dedicated square mount on the online camera panel of the control graphical user interface (GUI). A UV image is taken with a UV microscope prior to fast cooling. The silicon chip presented in the work of Mueller *et al.* (2015 ▸) and in further work (Oghbaey *et al.*, 2016 ▸; Owen *et al.*, 2017 ▸) has cavities for data collection on prepositioned crystals and fiducial marks at the corners. Oghbaey *et al.* (2016 ▸) implemented a UV–Vis spectrometry offline mapping of the wells of the same silicon chip to determine which wells were filled. Coordinate referencing between prelocation and measurement systems can also be performed by image alignment or feature matching (Sanchez-Weatherby *et al.*, 2019 ▸).

**Figure 1:**
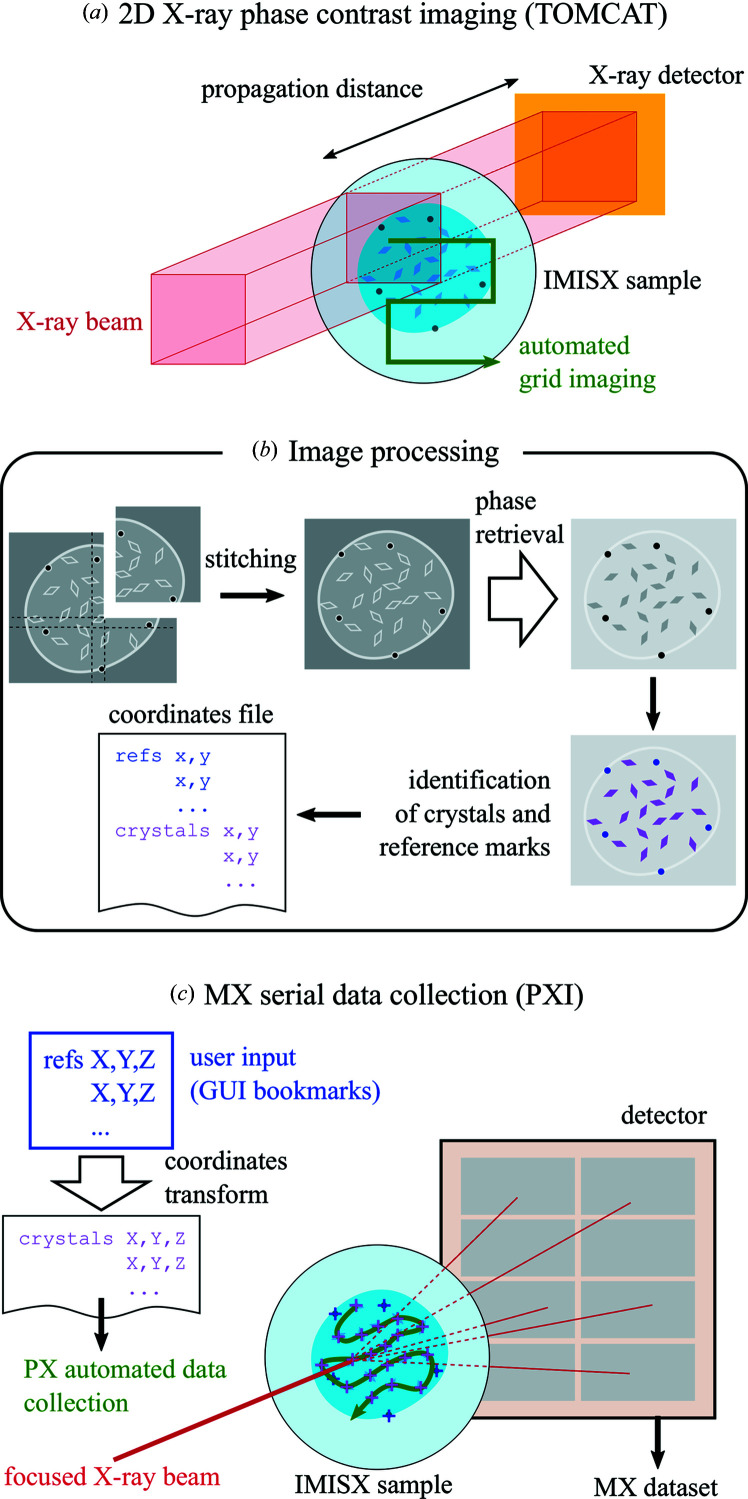
A schematic view of the workflow of the offline prelocation method based on 2D X-ray full-field phase-contrast imaging, as performed in this study. In (*a*), only the scintillator is represented as the X-ray detector and the schematic is approximately to scale. Operations performed in (*b*) and (*c*) are detailed in the main text. White arrows denote the main mathematical transformations.

Here we demonstrate how 2D full-field phase-contrast imaging can be used to prelocate crystals in double-sandwich *in meso in situ* serial X-ray crystallography (IMISX) samples (Fig. 1 ▸). Here, full field refers to an imaging method where the beam size is substantially larger than the crystals, as opposed to scanning methods using a beam focused to a size similar to or smaller than that of the crystals. Grid-wise imaging was used to image extended areas of the sample without compromising the imaging resolution. Coordinate transfer and MX data collection, of both rotation wedges and still series with various tilt angles, were performed based on the determined coordinates. X-ray imaging prelocation and MX data collection were carried out under cryogenic conditions to prevent any motion of the crystals within the sample, and to simplify sample storage and transfer. Two examples of protein crystals embedded in LCP were studied: lysozyme as a model soluble protein and a peptide transporter (PepT_St_) as a more challenging membrane protein. Steel beads were introduced in the samples to be used as fiducials for coordinate referencing. These metal marks can be detected – potentially automatically – at an MX beamline from their fluorescence signal, either using an available silicon drift detector at a synchrotron beamline or using a charge-integration detector like JUNGFRAU at an XFEL facility (Martiel *et al.*, 2020 ▸). Full-field phase-contrast X-ray imaging is a conventional imaging method that can be performed at most X-ray imaging beamlines (Paganin *et al.*, 2002 ▸). We believe that the demonstrated offline prelocation workflow could be implemented similarly in many other synchrotron facilities using already available equipment – under the reserve of beam time allocation at an X-ray imaging beamline or the availability of a modern microfocus X-ray laboratory source with sufficient coherence properties – and therefore benefits projects with high dose sensitivity.

## 2. Materials and methods   

### 2.1. Protein production, crystallization and sample preparation   

Lysozyme from chicken egg white and the peptide transporter, PepT_St_, from *Streptococcus thermophilus* were used in this study. Lysozyme was sourced from Sigma (St. Louis, MO, USA), and PepT_St_ was produced recombinantly in *Escherichia coli* and purified from biomass following published protocols (Lyons *et al.*, 2014 ▸). The LCP crystallization trials were performed by following the established protocol using two 100 µl Hamilton glass syringes and a coupler (Caffrey & Cherezov, 2009 ▸). Lysozyme-laden LCP was produced by mixing the 50 mg ml^−1^ lysozyme with mono­acyl­glycerol (9.9 MAG) in a volume ratio of 2 to 3. The PepT_St_-laden LCP was obtained by mixing the 10 mg ml^−1^ protein solution with 7.8 MAG in a volume ratio of 1:1.

For the IMISX plates setup (Huang *et al.*, 2015 ▸), cyclic olefin copolymer (COC) film with 25 µm thickness or silicon nitride windows with 1 µm thickness (Silson Ltd, Warwickshire, England) and double-stick spacer with 141 µm thickness were used. The 200 nl protein-laden LCP was dispensed manually into an *in situ* plate, covered with 1000 nl precipitant solution and then sealed. The precipitant solutions consisted of 0.5–1 *M* NaBr, 50–100 m*M* sodium acetate pH 4.5, 15–30%(*v*/*v*) polyethyl­ene glycol (PEG) 400 for lysozyme and 250–325 m*M* NH_4_H_2_PO_4_, 100 m*M* 4-(2-hy­droxy­ethyl)-1-piperazine­ethane­sulfonic acid (HEPES) pH 7.0, 21– 22%(*v*/*v*) PEG 400 for the PepT_St_ crystallization trial. Steel beads (Cospheric, Santa Barbara, USA, SSMMS-7.8 1–22 µm stainless steel metal microspheres, 7.8 g cm^−3^) were included in some of the samples, typically 10–20 beads around each LCP sample, by including them in the precipitant. A bath-sonicated suspension of 3 µg beads in 60 µl of the precipitants mentioned above was prepared and 1000 nl were deposited on the 200 nl protein-laden LCP. *In situ* crystallization was performed at 20°C.

The lysozyme crystals with 30 µm in their largest dimension grew within 30 min, and the IMISX wells were retrieved, excess precipitant was aspirated and the samples were mounted on a Y support (Huang *et al.*, 2018 ▸). The PepT_St_ crystals of 15–20 µm in size were observed after 24 h and the samples were mounted on a Y support using the same method as for lysozyme.

### 2.2. X-ray phase-contrast full-field imaging   

Propagation-based phase-contrast X-ray imaging was performed at the TOMCAT (X02DA) beamline of the SLS, Switzerland [Fig. 1 ▸(*a*)]. The X-ray source is a 2.9 T super­bending magnet, providing a photon source of 140 (*h*) × 45 µm (*v*) full width at half-maximum. Using a multilayer monochromator, a monochromatic X-ray beam of 18 keV energy was extracted with a flux of 1 × 10^12^ partially coherent photons s^−1^, shaped by slits in a rectangle of essentially homogeneous X-ray illumination, also called a top-hat beam. Partial coherence is a requirement for phase-contrast edge-enhancement imaging. The transverse coherence length was estimated to be 25 µm vertically and 5 µm horizontally (Lovric *et al.*, 2014 ▸), which is sufficient for the investigated samples. Samples were placed at a distance of 25 m from the photon source on a motorized stack of translation stages for positioning with an additional rotation axis to compensate for sample tilt (see Fig. S1 in the Supporting information). A horizontally placed Cryojet 5 (Oxford Instruments) was used to control the sample temperature to 100 K with the nozzle end placed ∼7 mm away from the sample, and center and shield flows set to 9.0 and 9.5 l min^−1^, respectively. X-ray images were collected by a 18 µm thick GGG scintillator placed 190 mm downstream from the sample, imaged with a 20× microscope and a 2560 × 2160 pixel camera (PCO.EDGE 5.5), covering a field of view of 702 × 832 µm at the sample position [red square in Fig. 1 ▸(*a*)]. The effective pixel size (side of the square) seen by the microscope was 0.325 µm. Grid-scan imaging was performed in an automatized manner synchronizing stage motion, camera recording and shutter opening. After a coarse alignment of the sample in the beam, the following images were collected: ten dark images (with shutter closed), ten flat-field images (with the sample fully moved out of the beam while keeping it cryogenically cooled by moving 5 mm towards the cryojet) and four images on each grid position on the sample. Grid positions are set so that images overlap by 10% to allow stitching. 3 × 3 or 3 × 4 grids were sufficient to cover the whole LCP sample, of ∼1.5 mm in its largest length, even coarsely aligned. The recorded exposure time per image was 50 ms. To avoid shutter synchronization issues, for each series of ten or four images the shutter was opened 20 ms before the first image and closed 20 ms after the last image, resulting in a total of 240 ms exposure to the X-ray beam per grid position, of which 200 ms were recorded by the detector. Final images were obtained by averaging within each series of ten or four images and performing flat-field correction as follows 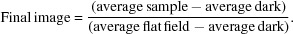



### 2.3. Image analysis   

The image-processing workflow is schematically presented in Fig. 1 ▸(*b*). Images were stitched in *ImageJ* 1.50i (Schneider *et al.*, 2012 ▸) using the included plugin for grid-wise or collection stitching (Preibisch *et al.*, 2009 ▸), which uses the Fourier transform phase-correlation method (Kuglin & Hines, 1975 ▸) to find translational offsets between sets of 2D or 3D images and is included in the standard distribution of *ImageJ*. Alternatively, the pair-wise registration plugin *MosaicJ* (Thévenaz & Unser, 2007 ▸) was also used successfully but it appeared less convenient because it required a manual coarse placement of the tiles prior to stitching. The stitched images were cropped around the region of interest (LCP sample). In some images, residual stripes appeared in spite of the flat-field correction. Therefore, a Fourier transform filter was applied to suppress frequencies lower than 40 pixels, as an empirical cutoff. Although images were already interpretable by eye thanks to the phase-contrast edge-enhancement effect around the crystals, a phase-retrieval step was performed following Paganin *et al.* (2002 ▸) in order to ease the automation of crystal detection (see Section S2 in the Supporting information). After phase retrieval, crystals appeared as distinct solid objects of the expected shape, in moderate contrast to the background. The spherical metal beads are easily identifiable thanks to the strong absorption from the metal which gives a strong contrast. Finally, images were converted into binary images with different thresholds for the detection of crystals or beads. A particle search was performed within the region of interest (LCP sample) using the built-in functionality in *ImageJ*, setting appropriate ranges in particle area (in pixels) and sphericity. The particle area was set to match the expected sizes of the crystals, taking into account the effective pixel size of 0.325 µm. The sphericity was used to exclude artefacts (sphericity < 0.2) and to discriminate crystals from metal beads (sphericity > 0.9). A list of results was exported as a list of 2D coordinates in the prelocation image plane for crystals or metal beads. References were either the positions of metal beads or easily identifiable features – such as silicon nitride window corners or bigger crystals – in the absence of metal beads.

### 2.4. Automatic serial crystallographic data collection   

Samples were transferred in cryogenic condition to the PXI beamline (X06SA) of the SLS and mounted on the standard goniometer endstation [Fig. 1 ▸(*c*)]. Three-dimensional beamline motor coordinates for the reference marks (or a subset of them if not all of them are visible) were extracted with the help of the bookmark feature (commonly used for pseudo-helical data collection) in the user interface *DA*+ (Wojdyla *et al.*, 2018 ▸), in which the user manually selects positions of interest by mouse clicking. The scan-request message sent by *DA*+ contains the bookmark coordinates to be used as reference marks. The geometrical transform between corresponding reference marks in the beamline space and prelocation image plane was determined using the *transformations.py* package (Gohlke, 2006 ▸) written by Christoph Gohlke. This package is a *Python* library for calculating 4 × 4 matrices for various geometrical transformations of arrays of 3D homogeneous coordinates, including translation, rotation, scaling and superimposition, as well as for converting between rotation matrices. In particular, the functions *superimposition_matrix* and *decompose_matrix* of the *transformations.py* package were used to determine the rotation angles linking the coordinates of the reference marks in the image plane and beamline space. The rotation matrix was recomposed using the *compose_matrix* function of the *transformations.py* package and applied to the coordinates of the crystals in the image plane to obtain coordinates in the beamline space. Further details can be found in Section S3. A new scan-request message was built with the calculated crystal coordinates and passed to the automatic data-collection routine as if it resulted from a raster-location process in the serial data-collection software *CY*+ (Basu *et al.*, 2019 ▸). On each sample, a series of still images with a given tilt angle were first collected for all crystal positions. The tilt angle was applied by adding an ω offset to the coordinates. Still images were collected with tilt angles 0, +5, +10, +15, −5, −10 and −15° on each crystal position with one image per tilt angle, with 0.1 s exposure per frame. The number of positions is given in Table 1 ▸. Specifically, for the COC IMISX lysozyme sample, seven still images with different tilts were collected at each of the 357 positions, *i.e.* 2499 still images in total. After collecting the still series, a rotation wedge (miniset) was collected at each of the same crystal positions, with 0.2° and 0.1 s exposure per frame and a total range of 15° per position for lysozyme (−7.5° to +7.5°), *i.e.* 75 frames per position. For SiN lysozyme, still-image series were collected at 233 pre­located positions, and the minisets with 15° rotation were collected from the first 137 positions. Two chips were used for the SiN PepT_St_ sample. Still-image series were collected from both chips with 922 positions, and minisets were collected from chip 1 only (474 positions) with a 10° total rotation per position (−5° to +5°), *i.e.* 50 frames per position. The sample-to-detector distance was set to 200 mm. The beamline was set up to an energy of 12.4 keV and a beam size of 20 (*h*) × 10 µm (*v*). The full flux of 1.3 × 10^12^ photons s^−1^ was used to collect all the datasets.

### 2.5. MX data processing and structure solution   

Still series were processed with the *CrystFEL* suite (White *et al.*, 2012 ▸, 2016 ▸) version 0.6, with the unity model in *partialator* (Table 1 ▸). Rotation wedges were processed with *XDS* and *XSCALE* (Kabsch, 2010 ▸) using an automatic serial crystallography pipeline (Basu *et al.*, 2019 ▸). A resolution cutoff in CC_1/2_ of ∼0.3 was manually applied to all datasets. Structures were phased by molecular replacement using *Phaser* (McCoy *et al.*, 2007 ▸) with PDB entries 5d5f and 5d58 (Huang *et al.*, 2016 ▸) as search templates for lysozyme and PepT_St_, respectively. *Phenix.refine* (Afonine *et al.*, 2012 ▸) was used to refine all structures. Data-collection parameters and refinement statistics are reported in Table 1 ▸. Dose values were calculated using the program *RADDOSE-3D* (Zeldin *et al.*, 2013 ▸).

## 3. Results and discussion   

### 3.1. Prelocation by 2D full-field X-ray imaging   

**Figure 2:**
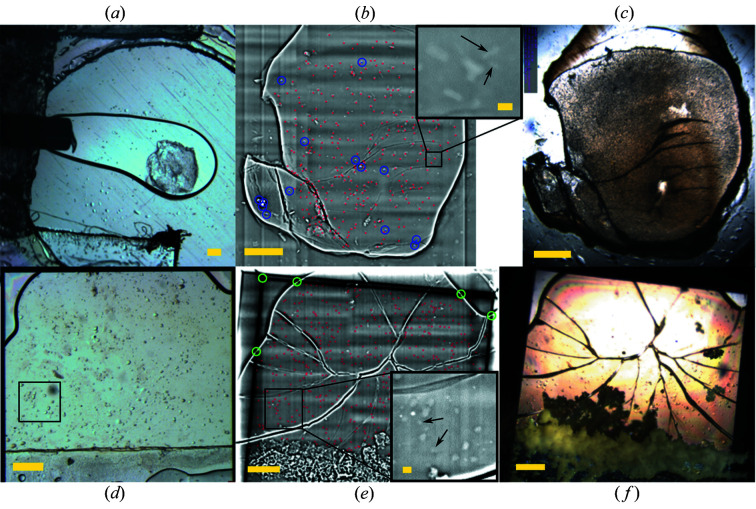
Representative images for lysozyme in COC with metal beads (*a*)–(*c*) and PepT_St_ in silicon nitride without metal beads (*d*)–(*f*). (*a*), (*d*) Optical-microscopy views before cryo-cooling. (*b*), (*e*) Edge-enhanced X-ray images from TOMCAT. (*c*), (*f*) Views with the online microscope at PXI. The online viewing images were rotated and mirrored as needed for clarity. Colors in (*b*) and (*e*) show the positions of crystals (red dots) and metal beads (center of the blue circles) from the particle search, and easily identifiable references in the absence of metal beads (center of the green circles). The scale bars are 200 µm, and 20 µm in the insets of (*b*) and (*e*). The arrows in (*b*) and (*e*) show some examples of the smallest identifiable crystals. The squared area in (*d*) corresponds to the inset in (*e*). Cracks in (*e*) and (*f*) result from the freezing of the mesophase between the rigid silicon nitride windows.

Figs. 2 ▸(*b*) and 2 ▸(*e*) show representative examples of lysozyme and PepT samples imaged by full-field phase-contrast X-ray imaging. Identical imaging parameters were used for all samples, after a tuning of parameters (energy between 18 and 22 keV, distance between 16 and 28 mm, and recorded exposure time between 150 and 250 ms) to optimize contrast and resolution (Fig. S2). The example of lysozyme essentially shows a visually opaque LCP sample in the online viewing of the PXI beamline [Figs. 2 ▸(*c*) and S5], where the largest metal beads can be seen but not the crystals. In the X-ray images, crystals appear as objects of moderate contrast, resulting from the edge-enhancing phase contrast of free-space propagation of the X-ray beam after the sample (Paganin *et al.*, 2002 ▸). Crystals of a few micrometres in size can be identified [Fig. 2 ▸(*b*) inset, where a 5 × 10 µm crystal is clearly identified], although they were not necessarily included in the automatic crystal selection in these experiments. Fourier ring correlation analysis with a half-bit threshold criterion (van Heel & Schatz, 2005 ▸) between overlapping areas of adjacent image grid tiles yielded, in this case, a maximum resolution of 4.76 pixels, *i.e.* 1.54 µm (Fig. S7). Metal beads appear as round objects of strong contrast, thanks to the added absorption contribution to the contrast. A residual phase-contrast halo was still observed around the metal beads owing to the semi-empirical tuning of the phase-retrieval step being targeted on the crystals (Fig. S4), but this does not interfere with their automatic selection thanks to the sufficient contrast. Imaging parameters could be tuned to adapt to samples with different resolution requirements, in particular with respect to the crystal size (Fig. S2). The pixel size is related to the field of view, *i.e.* smaller pixels result in smaller grid tiles. Higher resolutions, which are generally correlated to smaller pixels, require higher doses and more work at the stitching step since more tiles are needed to cover the same total area on the sample.

A semi-automatic particle search was performed within the LCP area, easily identified through its sharp edges. Some wrinkles or cracks in the mesophase owing to the flash-cooling process are observed, in particular in the silicon nitride sandwich samples, which behave as rigid walls [as, for example, in Figs. 2 ▸(*e*) and 2 ▸(*f*)]. However, the cracks delimit continuous areas of smooth background where the semi-automatic particle search was possible. This smooth background directly results from the nearly constant or slowly varying thickness of the flat double-sandwich samples used. Two-dimensional projections recorded on samples without enclosure, such as the scooped LCP sample investigated by Polikarpov *et al.* (2019 ▸), display a noisier background which would make crystal finding based on single 2D projections more challenging. Segmenting of 3D tomograms has then been used instead (Polikarpov *et al.*, 2019 ▸). Although in our work, 2D imaging is used as an offline prelocation method at a separate imaging beamline, in the case of MX beamlines possessing full-field X-ray capabilities such as the PETRA III P14 beamline, this method could readily be used as an online crystal-location method on flat samples. In our proof-of-principle experiment, the crystal search was a simple particle search after thresholding and binarization, with the size and sphericity as selection criteria. However, other more sophisticated detection parameters can be used, such as feature matching or other shape-specific search methods for crystals of characteristic shape.

Another point of discussion is the time overhead caused by the imaging and image analysis to extract crystal coordinates. In the case of our proof-of-principle work, methods had to be developed and parameters had to be optimized at all steps, which took time. However, many of the imaging and image-processing parameters are expected to be transferrable to similar samples, thus allowing automation of the imaging, phase-retrieval and particle-search steps. The computer calculations themselves (stitching, particle search, coordinate transfer) take less than a minute for one sample on a standard laptop.

The access to an X-ray imaging beamline, such as the SLS TOMCAT beamline we have used in this study, remains an open point. Arranging two beam times for such experiments may represent a difficulty, but the use of cryo-cooled samples makes it possible to separate the imaging and MX data-collection steps in time, and ensure that the crystals do not move between these two steps and that registration is not lost. Alternatively, modern laboratory-based X-ray sources with small photon-source sizes and therefore sufficient spatial coherence for edge enhancement could be used, as reported in the literature for a variety of imaging methods (Bidola *et al.*, 2017 ▸; Hauser *et al.*, 2014 ▸; Müller *et al.*, 2017 ▸; Romell *et al.*, 2018 ▸; Zhou *et al.*, 2015 ▸). One could, in principle, think of installing such a dedicated X-ray source either next to or at an MX beamline, or use laboratory equipment. The 2D X-ray imaging configuration could also be integrated by design into the MX measurement setup for online prelocation (Polikarpov *et al.*, 2019 ▸).

### 3.2. MX data collection   

The overall efficiency of the prelocation process was primarily measured as the final indexing success rate of the MX datasets finally collected in an automatic manner on the prelocated samples of lysozyme and PepT_St_ (Table 1 ▸). High indexing rates were obtained for all samples and datasets, which supports the robustness of the method. However, the best indexing rate did not exceed 75%. This result calls for discussion, and points to possible improvements of the current procedure. This overall efficiency of the prelocation process, measured as the final indexing success rate of the MX data, depends on several factors, which can be setup related or sample related. Unfortunately, in the final indexing rate measured on protein samples, all these inaccuracy factors are difficult to distinguish.

The most important setup-related factors are the reliability of the determination of crystal coordinates in the X-ray phase-contrast image at the prelocation step and the accuracy of positioning at the MX data-collection step. By reliability, we mean here whether a feature marked as a crystal at the detection step, and exported in the coordinate list, really corresponds to a crystal, or to some artifact or non-crystalline object. This reliability is reduced by the non-specificity of X-ray imaging to protein or crystalline material. Too strong background variations, such as the residual stripes visible in the case of PepT_St_ [Fig. 2 ▸(*e*)], also interfere with the simple thresholding method used here and result in a number of false crystal positions. Another related point is the number of crystals missed by the crystal-detection step applied on the prelocation image. A more conservative detection (here for instance, selecting larger objects) may have a higher reliability of its coordinates, but this may come at the expense of the number of diffraction images collected on a given sample, *i.e.* the overall data-collection efficiency.

The other main setup-related factor, the accuracy of crystal positioning, was studied separately using the fluorescence signal of an ideal model sample, a flat silicon nitride membrane bearing micrometre-sized nanofabricated metal objects (Martiel *et al.*, 2020 ▸). Thanks to this well characterized non-protein model sample, all other causes of inaccuracy can be eliminated. Detailed results are presented and discussed in the Supporting information (Section S1, Fig. S3). Still series with different tilt angles were collected. The best precision is observed at low tilt while higher tilt angles show a loss of precision in positioning. This phenomenon was also observed in the indexing rate of tilt series from protein samples (see Table S1 in the Supporting information). It can be interpreted as a centering imperfection, where the ω rotation axis is not placed exactly where the crystal is. This is more likely to be attributable to imperfect coordinate handling and transformation, rather than to the beamline hardware and controls, which are reported to be highly precise (Fuchs *et al.*, 2014 ▸; Wojdyla *et al.*, 2018 ▸). The imperfection of coordinate transformation might result from an imperfect recording of the 3D positions of the reference fiducials via the user GUI, for instance they may not be perfectly lying on a plane, or lying on a slightly tilted plane, or even imprecisely located on the right plane. This could cause a distorted mapping of the 2D prelocated coordinates in the 3D beamline space.

Sample-related factors comprise, for instance, the flatness and thickness of the sample, its integrity, and the diffraction properties of the crystals. The departure from an ideally flat geometry, as in the case of the COC sandwich where a bending of the sample can sometimes be observed (Huang *et al.*, 2018 ▸), also decreases the reliability of the coordinates. This could explain why the COC lysozyme sample presents a slightly worse indexing rate compared with the silicon nitride lysozyme sample (Table 1 ▸). Another source of inaccuracy at higher tilt or rotation angles is the fact that 2D imaging neglects the thickness dimension of the sample. Crystals placed off-plane therefore drift more easily out of the beam axis when the sample is rotated. This, in addition to the imperfect coordinate transformation mentioned above, could contribute to the worse indexing rates observed in the still series compared with the wedges datasets, since the maximum tilt angle used in stills was larger than for wedges. The angles and sample thicknesses used should therefore stay moderate, for instance a dozen degrees, as is generally the case in serial data collection in double-sandwich samples. The systematic difference between indexing rates of stills and wedges could also be linked to the different indexing methods and software suites used, and to the fact that still images contain fewer reflections than rotation frames. Especially in the case of silicon nitride enclosed samples, detachment of part of the frozen LCP sample where crystals had been located [Fig. 2 ▸(*f*)] caused a decrease in indexing rate, as, for simplicity, these positions were not excluded from the coordinate list. Crystal-specific properties such as the size and diffraction quality of individual crystals also influence the indexing process and therefore the measured overall efficiency. This could explain why the PepT_St_ samples yielded generally lower indexing rates than lysozyme.

### 3.3. MX data analysis   

The prelocation method was successfully applied to both the conventional MX data collection with rotation method and still-image data collection by serial crystallography. The X-ray data and refined structures are of good quality (Table 1 ▸) and did not display any sign of radiation damage, as seen for instance from the intact di­sulfide bridges of the lysozyme structures (Fig. S6). Di­sulfide bonds are the regions of the investigated proteins that are most sensitive to specific radiation damage, which occurs at an even lower dose than global radiation damage (Burmeister, 2000 ▸).

In the COC lysozyme case, the diffraction data quality between rotation wedges and still series is very comparable as judged by CC_1/2_ of merged data (Table 1 ▸). The *I*/σ values are very different between rotation and still data. However, they cannot be compared directly because the underlying error model and σ estimation are very different in data processing. Both structures were refined to similar *R*_work_/*R*_free_ values. In the SiN lysozyme case, the rotation data reached a higher resolution (1.6 Å) and the corresponding structure was refined to better quality with lower *R*_work_/*R*_free_. In this case, maybe the fiducial marking and coordinates transform were not very precise, which could result in crystal misalignment for stills at a higher tilting angle. Another possible explanation is that the crystals from the 137 positions only used for rotation data collection have better diffraction quality on average compared with all crystals from the 233 positions. The diffraction resolution of the SiN PepT_St_ sample is slightly worse for the rotation data. We attribute this difference to the crystal-quality variation between chip 1 and chip 2, as only chip 1 was used for rotation data collection. Such sample-to-sample variation is common for membrane-protein microcrystals.

### 3.4. Dose considerations   

The dose received by crystals in the 2D full-field phase-contrast imaging step was ∼100 Gy. This value is comparable to the 2D projections reported by Polikarpov *et al.* (2019 ▸), and much lower than doses typically absorbed by a crystal during raster-diffraction location, which is on the order of 100 kGy at 100 Hz (0.01 s per frame) with the full beam at the PXI beamline. In the grid imaging with stitching, crystals situated in edge overlaps receive twice the dose and crystals in corners receive four times the dose, however this represents only a minor part of the area. This effect could be reduced by increasing the imaging field of view at the expense of imaging resolution, in a compromise approach. The dose can also be reduced by using a higher X-ray energy at the expense of contrast (Fig. S2).

According to the dose-fractionation theorem, the dose required to retrieve information on a particular feature in 3D or 2D is in principle the same, meaning that projections with no apparent signal can be assembled in an exploitable 3D reconstruction (McEwen *et al.*, 1995 ▸). In the case of a flat sample geometry, we showed that a single exposure angle is directly exploitable for extracting the crystal coordinates, leading to a dose applied to the crystals that was unprecedentedly low for an X-ray-based crystal-prelocation method. It would be interesting to compare this 2D method with 3D tomograms acquired with a similarly low overall dose.

The protocol and dose used for MX serial data collection of wedges is conventional and independent from the prelocation process. Interestingly, in this study, the dose of the stills data-collection protocol with tilts is almost ten times lower than that for the wedges, with, for instance in the lysozyme case, 5 MGy at each position for rotation, versus 0.7 MGy at each position for stills. The dose for stills data collection could be further reduced by using fewer still exposures per position on a larger number of positions or samples. Such a protocol could have an advantage for challenging projects with dose-sensitive targets such as membrane proteins or metalloproteins, as long as the crystal supply is sufficient. Low-dose stills data-collection protocols benefit particularly from a low-dose crystal-location process such as the 2D X-ray imaging method demonstrated here.

### 3.5. Comparison with other prelocation techniques   

**Figure 3:**
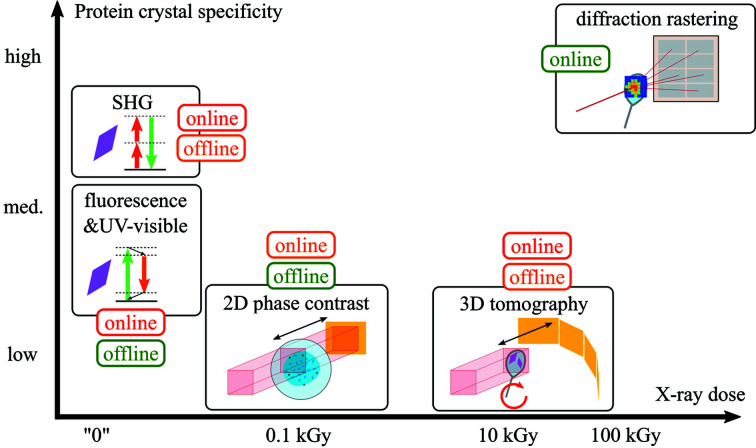
Various physical techniques for prelocation of protein crystals, as a function of their protein crystal specificity and absorbed X-ray dose. The colored boxes qualitatively illustrate the current or near-future general user accessibility as online or offline techniques: red denotes a low or difficult availability (*e.g.* a single beamline worldwide with difficult integration constraints in beamline design), orange denotes moderate availability (a few beamlines or laboratories) and green denotes an ubiquitous method or easy accessibility at most facilities.

Fig. 3 ▸ shows the qualitative positioning of 2D X-ray imaging compared with other possible prelocation techniques, in terms of dose applied and protein crystal specificity. An assessment of the current or near-future user accessibility of the method as an online or offline technique is also provided with color coding. Diffraction-based rastering is readily available at most beamlines and is the most specific of all available methods. However, it comes at a high dose cost in comparison with other methods – although the applied dose remains small compared with the dose typically applied during the final data collection, generally on the order of MGy to tens of MGy.

Optical methods do not apply any X-ray dose in principle, but their crystal specificity and accessibility vary. Tryptophan fluorescence and UV–Vis microscopy are widely available in crystallization laboratories and can serve as offline prelocation methods (Barnes *et al.*, 2018 ▸). However, integrating these techniques as online methods poses a challenge for the design of an inline optical system compatible with a wide range of light wavelengths (Newman *et al.*, 2016 ▸). Moreover, false positives for crystal detection do exist, such as protein-covered salt crystals, or undetectable cases such as proteins without any tryptophan residue. SHG is in principle very specific for protein crystals, with the exception of non-chiral space groups. However, its high cost versus gain ratio limits the availability for offline use in laboratories. Only one beamline worldwide is equipped with SHG online imaging. X-ray-based phase-contrast methods rely on the difference of optical index between the crystal and its environment, which generates a phase contrast, but they are not protein crystal specific as other non-crystalline objects can induce the same contrast, or even a more pronounced one.

To date, only a few MX beamlines can perform a fast switch between a microfocused configuration for diffraction data collection and a large-area flat beam for imaging, which limits for now the online availability of X-ray imaging for crystal detection, at least for 3D tomography (Polikarpov *et al.*, 2019 ▸). Simple 2D phase-contrast imaging, used in this work, has less stringent hardware requirements compared with 3D tomography, since the sample is not rotated, data analysis is simpler and the applied dose is a fraction of the dose required for a 3D tomogram recorded employing typical data-acquisition schemes. In addition, the possibility to perform grid-wise 2D imaging allows for smaller fields of views and higher resolution to be used, and therefore eases the requirements for the X-ray camera system for online prelocation. A prerequisite for direct prelocation of crystals in 2D projections is, however, that the sample thickness varies slowly in space, as in the flat samples investigated here. Propagation-based inline phase-contrast imaging (Snigirev *et al.*, 1995 ▸) requires only a partially coherent ideally flat profile beam over the imaged area and no optical elements like condenser or objective lenses. It is thus available at imaging beamlines in most synchrotron facilities and opens up possibilities for offline prelocation using an imaging beamline onsite or a modern laboratory X-ray source.

## 4. Conclusions   

We have produced an end-to-end demonstration of a simple yet robust and efficient offline crystal-prelocation method based on 2D full-field phase-contrast X-ray imaging on flat double-sandwich samples, a well established format for serial crystallography. The dose applied to the samples was particularly low for an imaging resolution on the order of a micrometre. This method represents an alternative to commonly used diffraction-based rastering techniques, thanks to its very low dose. For comparable performance, its hardware requirements are reduced compared with advanced imaging techniques such as 3D X-ray tomography or SHG optical methods, which could increase its accessibility for users. Our results are not only of relevance for synchrotron serial crystallography, but also for fixed-target SFX at XFELs.

## Supplementary Material

Supporting information. DOI: 10.1107/S2052252520013238/ro5023sup1.pdf

PDB reference: lysozyme from SiN IMISX setup collected by rotation serial crystallography, 6yod

PDB reference: lysozyme from SiN IMISX setup collected by still serial crystallography, 6yoe

PDB reference: PepTSt from COC IMISX setup collected by rotation serial crystallography, 6yof

PDB reference: lysozyme from COC IMISX setup collected by rotation serial crystallography, 6yob

PDB reference: lysozyme from COC IMISX setup collected by still serial crystallography, 6yoc

PDB reference: PepTSt from COC IMISX setup collected by still serial crystallography, 6yog

## Figures and Tables

**Table 1:** Data-collection parameters and refinement statistics Values in parentheses are for the highest-resolution shell, unless indicated otherwise.

	Lysozyme				PepT_St_	
Support	COC IMISX		Silicon nitride sandwich		Silicon nitride sandwich	
PDB code	6yob	6yoc	6yod	6yoe	6yof	6yog
Data-collection mode	Rotation wedges	Still series with tilt	Rotation wedges	Still series with tilt	Rotation wedges	Still series with tilt
No. of samples used	1	1	1	1	1	2
No. of positions collected	357	357 × 7	137	233 × 7	474	922 × 7
Indexing success rate†	263/357 = 73.7%	1511/2499 = 60.5%	114/137 = 75%	1051/1631 = 64.4%	322/474 = 68.0%	3384/6454 = 52.7%
No. of merged images	8250 images from 110 wedges	1511 still images	4500 images from 60 wedges	1051 still images	7350 images from 147 wedges	3384 still images
Space group	*P*4_3_2_1_2	*P*4_3_2_1_2	*P*4_3_2_1_2	*P*4_3_2_1_2	*C*222_1_	*C*222_1_
Unit-cell parameters						
*a*, *b*, *c* (Å)	79.17, 79.17, 38.24	78.70, 78.70, 37.79	78.99, 78.99, 38.19	78.50, 78.50, 40.46	102.45, 110.7, 111.18	102.43, 110.7, 111.28
α, β, γ (°)	90, 90, 90	90, 90, 90	90, 90, 90	90, 90, 90	90, 90, 90	90, 90, 90
Resolution (Å)	39.60–1.85 (1.89–1.85)	55.65–1.85 (1.89–1.85)	39.49–1.60 (1.64–1.60)	55.51–1.85 (1.89–1.85)	46.52–2.45 (2.51–2.45)	75.18–2.30 (2.39–2.3)
*R*_meas_ or *R*_split_ ‡	0.25 (7.79)	0.24 (0.95)	0.21 (12.17)	0.26 (1.20)	0.47 (70.4)	0.17 (2.15)
〈*I*/σ(*I*)〉	7.89 (0.52)	3.59 (1.32)	13.58 (0.86)	2.30 (0.85)	13.01 (1.34)	3.73 (0.93)
Completeness (%)	99.4 (94.4)	100 (100)	99.7 (96.9)	100 (96.4)	100 (100)	100 (99.95)
Multiplicity	17.44 (15.4)	31.10 (16.7)	40.98 (36.73)	11.29 (5.6)	52.17 (46.73)	51.90 (32.6)
CC_1/2_	0.99 (0.27)	0.70 (0.34)	0.99 (0.26)	0.90 (0.22)	0.99 (0.56)	0.92 (0.33)
Refinement						
Resolution (Å)	39.58–1.85	55.98–1.85	39.49–1.60	55.51–1.85	49.55–2.45	55.64–2.30
No. of unique reflections	10828	10612	16419	11268	23579	28440
*R*_work_/*R*_free_	0.21/0.24	0.22/0.24	0.19/0.21	0.26/0.28	0.22/0.27	0.24/0.27
No. of atoms						
Protein	1000	1000	1000	1000	3546	3475
Ligand/ions	19	19	29	21	463	463
Water	75	53	99	55	73	77
*B* factors (Å^2^)						
Protein	33.75	33.33	31.66	46.82	68.55	57.31
Ligand/ions	41.47	41.5	46.16	59.91	83.20	69.03
Water	36.45	35.85	39.14	48.15	59.97	48.79
R.m.s. deviations						
Bond lengths (Å)	0.008	0.005	0.016	0.003	00002	0.002
Bond angles (°)	0.90	0.68	1.38	0.46	0.55	0.55
Ramachandran plot						
Favored (%)	97.64	98.43	99.21	97.64	98.68	97.74
Allowed (%)	2.35	1.57	0.79	2.36	1.32	2.26
Outliers (%)	0	0	0	0	0	0
MolProbity clashscore	4.03	1.51	2.98	3.51	6.36	5.86

† The indexing success rate is given for stills as (number of images indexed by *CrystFEL*)/(total number of images collected) and for miniset wedges as (number of minisets indexed by *XDS*)/(total number of collected minisets).

‡ *R*_meas_ is given for rotation datasets and *R*_split_ is given for still-images datasets.
