# Supplementary material for: Low-dose in situ prelocation of protein microcrystals by 2D X-ray phase-contrast imaging for serial crystallography
Source: IUCrJ. 2020 Oct 23;7(Pt 6):1131–41. doi: 10.1107/S2052252520013238 (PMC7642777; doi:10.1107/S2052252520013238)
Supplement: Supplementary file 1 [file m-07-01131-sup1.pdf]

IUCrJ

Volume 8 (2021)

Supporting information for article:

Low-dose in situ prelocation of protein microcrystals by 2D X-ray phase-contrast imaging for serial crystallography

Isabelle Martiel, Chia-Ying Huang, Pablo Villanueva-Perez, Ezequiel Panepucci, Shibom Basu, Martin Caffrey, Bill Pedrini, Oliver Bunk, Marco Stampanoni and Meitian Wang

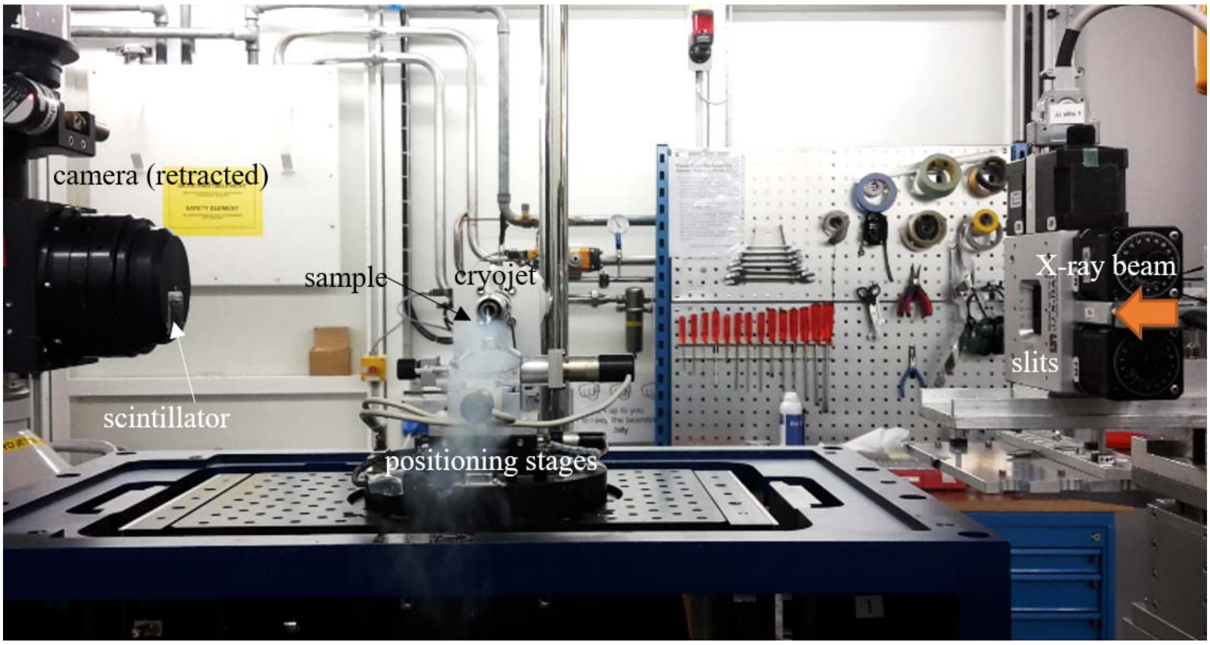

Figure S1 IMISX sample mounted under cryo-cooled conditions at the TOMCAT (X02DA) beamline of the SLS.

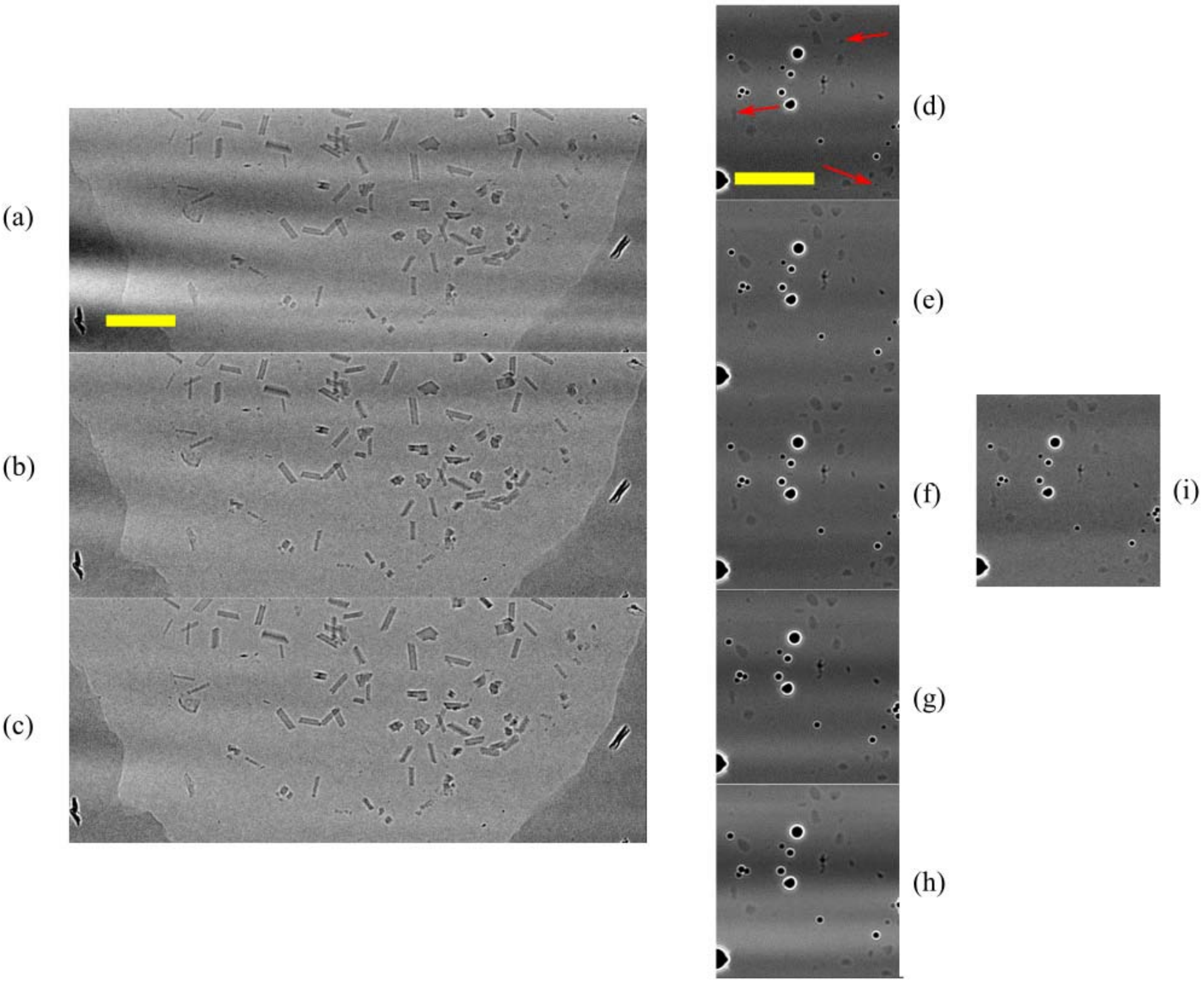

Figure S2 Example images to illustrate the optimization of imaging parameters, prior to phase retrieval. Scale bars are 100 µm. (a-c) Influence of the exposure time: (a) 150 ms, (b) 200ms, (c) 250 ms on a lysozyme sample in COC sandwich imaged with 13 cm propagation and 22 keV X-ray beam energy. The smoother, less noisy image is obtained with longer exposure times, making the interpretation easier. (d-h) Influence of the propagation distance on a PepT sample imaged at 18 keV with 4 exposures of 50 ms averaged: (d) 16 cm, (e) 19 cm, (f) 22 cm, (g) 25 cm, (h) 28 cm. With increasing propagation length, the contrast increases but small details (for example pointed by red arrows) are blurrier in (h) compared to (d). (i) Influence of the X-ray beam energy on the same PepT sample imaged at 22 keV with 4 exposures of 50 ms averaged, to be compared to (f). The contrast decreases with increasing X-ray beam energy, i.e. the contrast of all crystals is lower in (i) compared to (f).

S1. Precision characterization

The precision of the addressing of positions was investigated using fluorescence detection. The sample used was a microfabricated membrane carrying cross-shaped metal objects (Martiel et al., 2020 as referenced in the main text), of which the positions are perfectly known (with nanometer precision) from the fabrication process. The fluorescence was detected as reported in Martiel et al. 2020 (referenced in the main text), by summing counts in a region of interest of the EIGER 16M image. The fraction of fluorescence intensity was obtained by dividing the summed counts of each image by the summed counts in an image recorded from a position where no metal is present and summed counts are therefore minimal. Figure S3a shows the fluorescence signal resulting from a fine grid scan over a single object, which is expected to be the convolution of the object shape and beam profile. From this scan, one can see that a positioning error of 5 to 10 µm (vertically or horizontally) corresponds to a fluorescence signal of 30% of the maximum (thick black line).

Some of the objects were arbitrarily designated as reference fiducials and used as such in the automatic data collection process. Instead of crystal coordinates from a prelocation image, the design positions of the objects were used. Series of still images with various tilt angles were collected (Figure S3b&c). The fluorescence fractions obtained are worse for larger tilt angles.

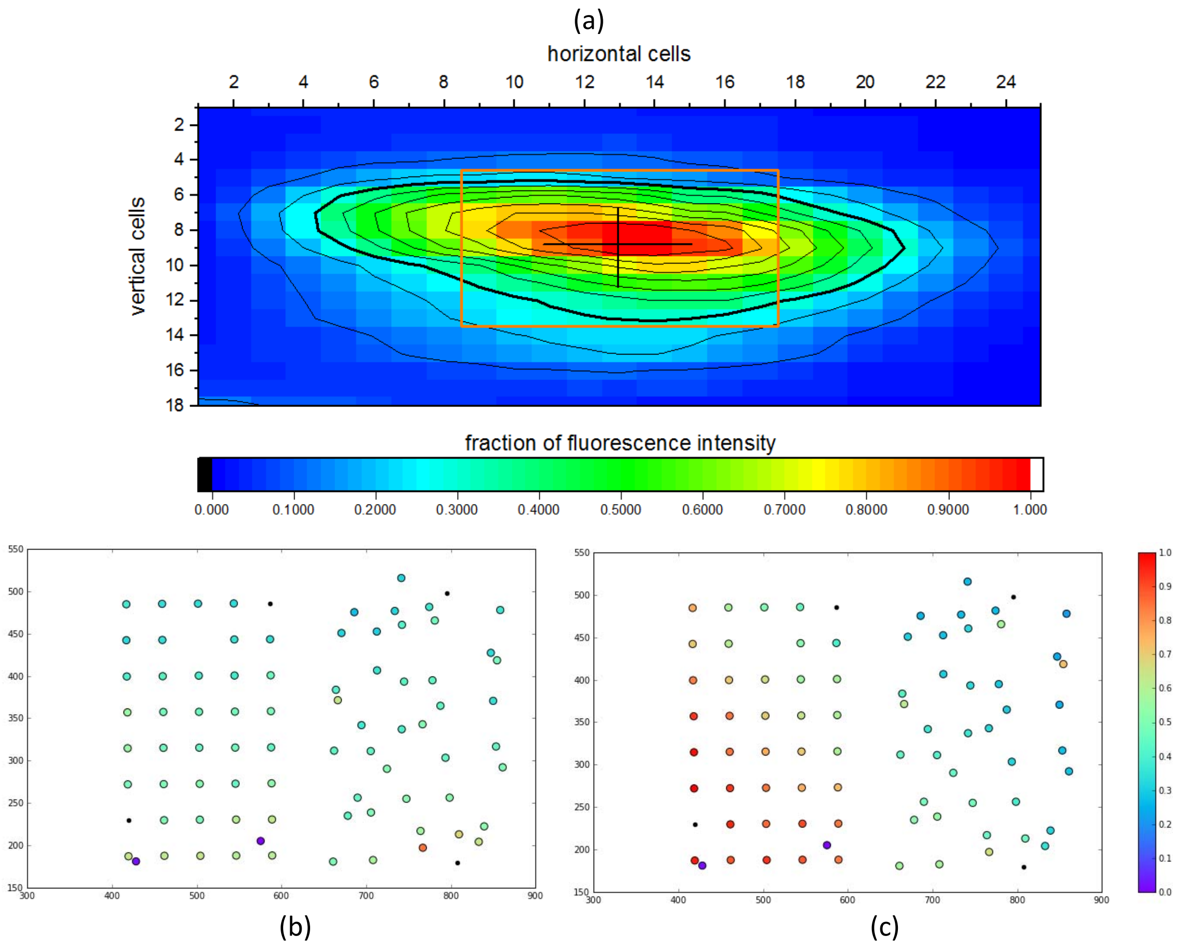

Figure S3 (a) Scan over a single metal object, with 2 µm x 1 µm cell size and 20 µm x 10 µm beam size. The beam box size is drawn in orange at the same scale. The thick black line shows a 30% fluorescence intensity, black lines are at 10% increment. (b, c) Examples of fluorescence intensity with -5 (b) and -15 deg (c). Two of the positions were added as control without metal, and appear here in purple since no fluorescence was detected at these positions. Fiducials are the small black dots.

Table S1 Variation of the measured fluorescence hit rate in the microfabricated metal model sample and indexing rate in a lysozyme sample as a function of the tilt angle applied for data collection.

Tilt angle (deg)	Metal sample (30% fluorescence threshold)	Lysozyme in COC sandwich: Percentage of images indexed
-15	0.84	51.82%
-10	0.93	56.58%
-5	1	62.18%
0	0.71	64.71%
5	0.63	62.75%
10	0.46	59.10%
15	0.41	53.50%

S2. Phase retrieval

The equation 12 from Paganin et al., 2002 (copied below) was implemented in python.

$$T(\mathbf{r}_\perp) = -\frac{1}{\mu}\log_e\left(\mathcal{F}^{-1}\left\{\mu\frac{\mathcal{F}\{M^2 I(M\mathbf{r}_\perp, z=R_2)\}/I^{in}}{R_2\delta|\mathbf{k}_\perp|^2/M+\mu}\right\}\right). \quad (12)$$

Definition of parameters:

```
mu = 0.001 # um-1
phase_shift = -0.4 # rad/um
E = 18 #keV
wavelength = 12.4/E/10000 # um
delta = -wavelength/(2*math.pi)* phase_shift
delta = 4.38560287631e-06 # result calculated for the parameters given
M = 1
R2 = 19 *1e4 # um
pixel_size = 0.325 # um
FOV_pix = img.shape
FOV_um = (FOV_pix[0] * pixel_size, FOV_pix[1] * pixel_size)
flux = 1e12 # ph/s
I_in = flux / (FOV_um[0] * FOV_um[1]) # ph/s/um2
```

```
def phase_retrieval_intensity_tranport(image, mu, delta, I_in, M, pixel_size, R2):
       '''calculates the projected thickness as in eq. 12 of Paganin et al., 2002, J.
Microscopy, from an image, the absoption coefficient mu (um-1), the real part of the deviation
of the refractive index from unity delta, the uniform intensity of the incident radiation I_in
(ph/s/um2), the magnification of the image from the point source illumination M, the pixel
size (um), the propagation distance R2 (um).'''
    F = fft.rfft2(image)
    k_x = 1/(pixel_size*image.shape[0])
    k_y = 1/(pixel_size*image.shape[1])
    #print k_x,k_y
    k_array = np.zeros((F.shape[0],F.shape[1]))
    A = np.zeros((F.shape[0],F.shape[1]), dtype=np.complex128)
    for i_k in range(k_array.shape[0]):
        for j_k in range(k_array.shape[1]):
            k_array[i_k,j_k] = np.sqrt((i_k * k_x)**2 + (j_k * k_y)**2)
            A[i_k,j_k] = mu * F[i_k,j_k] / (I_in * (R2 * delta * k_array[i_k,j_k]**2 / M +
mu))
    Fm1 = fft.irfft2(A)
```

```
    T = np.multiply(-1/mu, np.log(Fm1))
    return T
```

This function is applied to the original image and the flipped image, then both are added and normalized.

```
phase_image = phase_retrieval_intensity_tranport(img, mu, delta, I_in, M, pixel_size, R2)
phase_image_flipped = phase_retrieval_intensity_tranport(np.fliplr(img), mu, delta, I_in, M,
pixel_size, R2)
added_image = np.add(phase_image,np.fliplr(phase_image_flipped))
```

Figure S4 illustrates the effect of phase retrieval on the metal beads and a lysozyme crystal in COC sandwich. The difference of contrast between steel beads and crystals is very visible, as well as the effect of phase retrieval on the edge-enhancement curve.

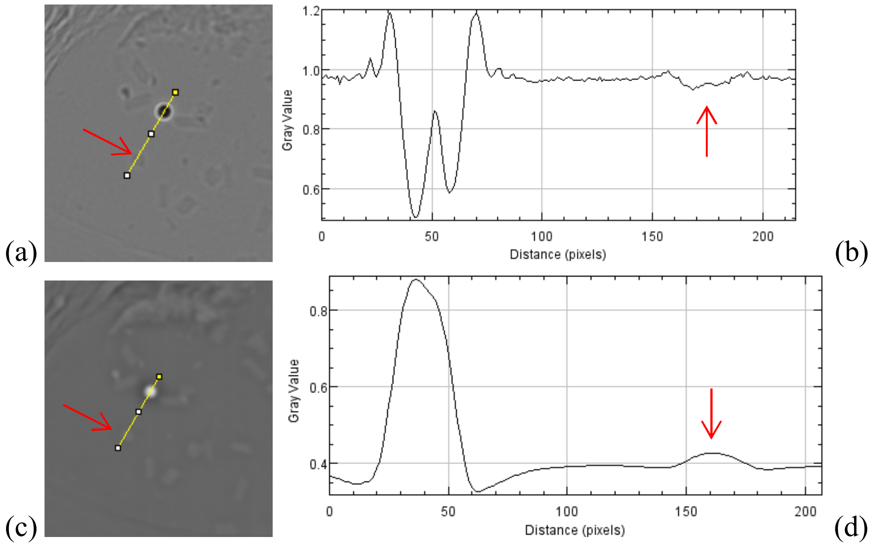

Figure S4 Steel bead and crystal in a lysozyme COC sample, before (a&b) and after (c&d) phase retrieval. (a) and (c) are details from X-ray images, (b) and (d) are plots of the gray level values along the yellow lines in (a) and (c), with black as low value and white as high value. The steel bead appears as a black object in (a) and as a white object in (c). In (b) and (d), the position of the steel bead correspond to the large variations of gray value. The position of the lysozyme crystal is shown by a red arrow in all panels. In (a) and (b), phase contrast oscillations at the edges are visible around the ojects, while these oscillations are essentially suppressed by the phase retrieval in (c) and (d).

S3. Coordinate transfer

The following workflow used for the proof-of-principle automated data collection:

1. mount sample
2. read prelocation data exported from ImageJ particle selection
   - fiducials
   - crystal coordinates
3. **connect the prelocation program to the daqserver queue to be ready to read the messages sent by DA+**
4. align sample **flat** by checking that the whole area is in focus
5. bookmark fiducials at highest zoom level (can zoom out for overview)
6. adjust parameters in DA+ GUI
7. in the "**Collection"** tab of DA+ check the box "**Split Dataset into Defined Bookmarks"**
8. click «**Start Data Acquisition» (**this will not start anything but will send the coordinates of the *picked* fiducials to this program)
9. Start the **DA+ server**
10. **disconnect the prelocation program from the daqserver queue**
11. run the python transformation code
12. create a new data collection message
13. start data acquisition by sending the message to DA+ server

The transformation code used can be found below, where transformations.py is the package provided by Christoph Gohlke (Gohlke, 2006 as referred in the main text):

```
import numpy as np
import transformations

def sort_y(coord):
    '''sorts an array of coordinates (np array of dimensions n,2) with respect to the
    last column y, keeping the pairs together'''
    y_sorted = coord[np.lexsort(coord.T)]
    return y_sorted

def sort_center_3d(coord):
    '''sorts an array of 3D coordinates by their distance to the current origin'''
    distances = np.zeros((coord.shape[0], 1))
    for i in range(len(coord)):
        distances[i, 0] = np.array([[dist_3d((coord[i, 0], coord[i, 1], coord[i, 2]), (0, 0,
0))]])
    sorted_coord = conversions.sort_y(np.concatenate((coord, distances), axis=1))
    return np.delete(sorted_coord, -1, 1)

def find_geo_center(coord):
    '''find the geometric center of a set of coordinates'''
    center = np.average(coord, axis=0)
    return center

def center_coord(coord, new_center):
    '''translates coordinates to set a new origin'''
    centered_coord = coord - new_center
    return centered_coord

# add a 3rd column to the 2D prelocation coordinates of the fiducials and crystals
preloc_refs_coord = np.concatenate((preloc_refs.as_matrix(columns=['XM', 'YM']),
                                    np.zeros((preloc_refs.as_matrix(columns=['XM',
'YM']).shape[0],1))),axis=1)
preloc_xtal_coord = np.concatenate((preloc_xtal.as_matrix(columns=['XM', 'YM']),
                                    np.zeros((preloc_xtal.as_matrix(columns=['XM',
'YM']).shape[0],1))),axis=1)
```

```
#find the center of mass (3d)
preloc_cm = find_geo_center(preloc_refs_coord)
bl_cm = find_geo_center(bl_refs)

# center the ref coordinates around their cm, sort them (3d)
sccc_bl_refs = sort_center_3d(conversions.center_coord(bl_refs, bl_cm))
sccc_preloc_refs = sort_center_3d(conversions.center_coord(preloc_refs_coord, preloc_cm))

# for the crystals, do only the centering (no sorting needed)
sccc_preloc_xtal = conversions.center_coord(preloc_xtal_coord, preloc_cm)

# scale the coordinates together, scaling the prelocated coords to the bl coords
sc_facts = []

for n in range(4):
    x1, y1, z1 = sccc_bl_refs[n]
    x2, y2, z2 = sccc_preloc_refs[n]
    sc_facts.append(np.sqrt(np.power(x1, 2) + np.power(y1, 2) + np.power(z1, 2)) /
np.sqrt(np.power(x2, 2) + np.power(y2, 2) +np.power(z2,2)))

scale_factor = np.average(sc_facts)
scaled_preloc_refs = sccc_preloc_refs * scale_factor
scaled_preloc_xtal = sccc_preloc_xtal * scale_factor

# use the transformations package to find the angles
transfo_mat_2 = transformations.superimposition_matrix(scaled_preloc_refs.T.tolist(),
sccc_bl_refs.T, scale=True, usesvd=True)
scale2, shear2, angles2, translate2, perspective2 =
transformations.decompose_matrix(transfo_mat_2)
angles_deg2 = [a * 180/math.pi for a in angles2]

# now transfer the prelocated coordinates (refs and crystals) into beamline space

# make a pure rotation matrix
test_transfo_mat = transformations.compose_matrix(scale=None, shear=None, angles=angles2,
translate=None, perspective=None)
print(test_transfo_mat)

# add 4th column of ones for the transformation
preloc_refs_w2 = np.concatenate((scaled_preloc_refs,
np.ones((scaled_preloc_refs.shape[0],1))),axis=1)
preloc_xtal_w2 = np.concatenate((scaled_preloc_xtal,
np.ones((scaled_preloc_xtal.shape[0],1))),axis=1)

# apply the pure rotation matrix and remove 4th column
rot_preloc_refs = np.delete(np.asarray(np.dot(test_transfo_mat,preloc_refs_w2.T)).T,-1,1)
rot_preloc_xtal = np.delete(np.asarray(np.dot(test_transfo_mat,preloc_xtal_w2.T)).T,-1,1)

# move back to the center of mass of the beamline coordinates
m_bl_cm = np.multiply(bl_cm, -1)

final_refs = center_coord(rot_preloc_refs, m_bl_cm)
final_xtal = center_coord(rot_preloc_xtal, m_bl_cm)
check_bl_refs = center_coord(sccc_bl_refs, m_bl_cm)
```

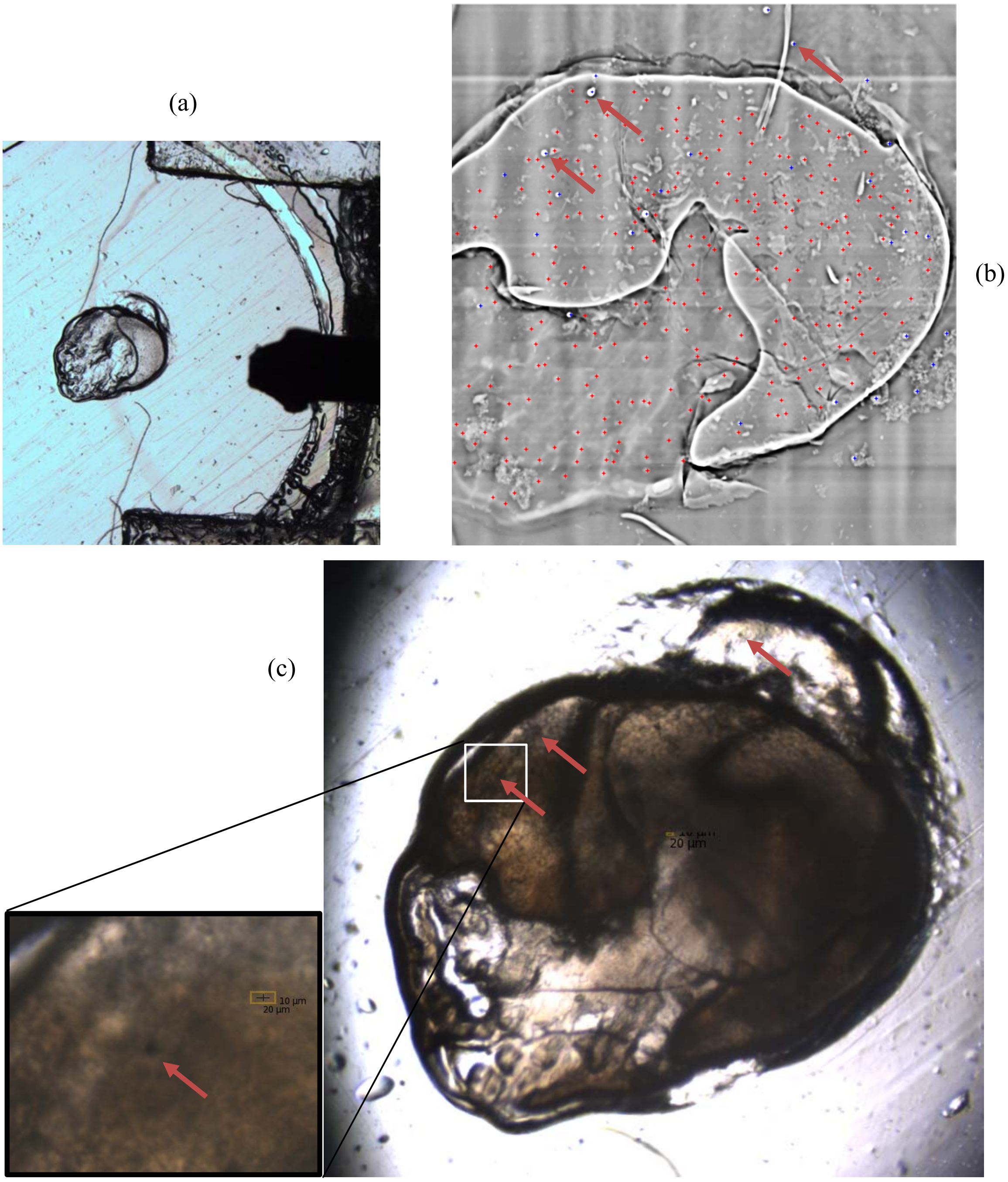

Figure S5 Examples for the visualization of some of the metal beads used as references in an essentially opaque lysozyme sample. (a) microscope picture before snap-cooling. (b) TOMCAT 2D phase-contrast prelocation image, with crystals and metal beads marked in red and blue respectively. (c) Online microscope picture at PXI at the lowest magnification, shown at the approximate scale of (b) for easier comparison. Bordeaux arrows indicate examples of metal beads. The inset shows the view at intermediate magnification. In the opaque bolus, only some of the large metal beads can be seen at low magnification. Increasing the magnification helped to visualize smaller metal beads.

(a) (b) (c)

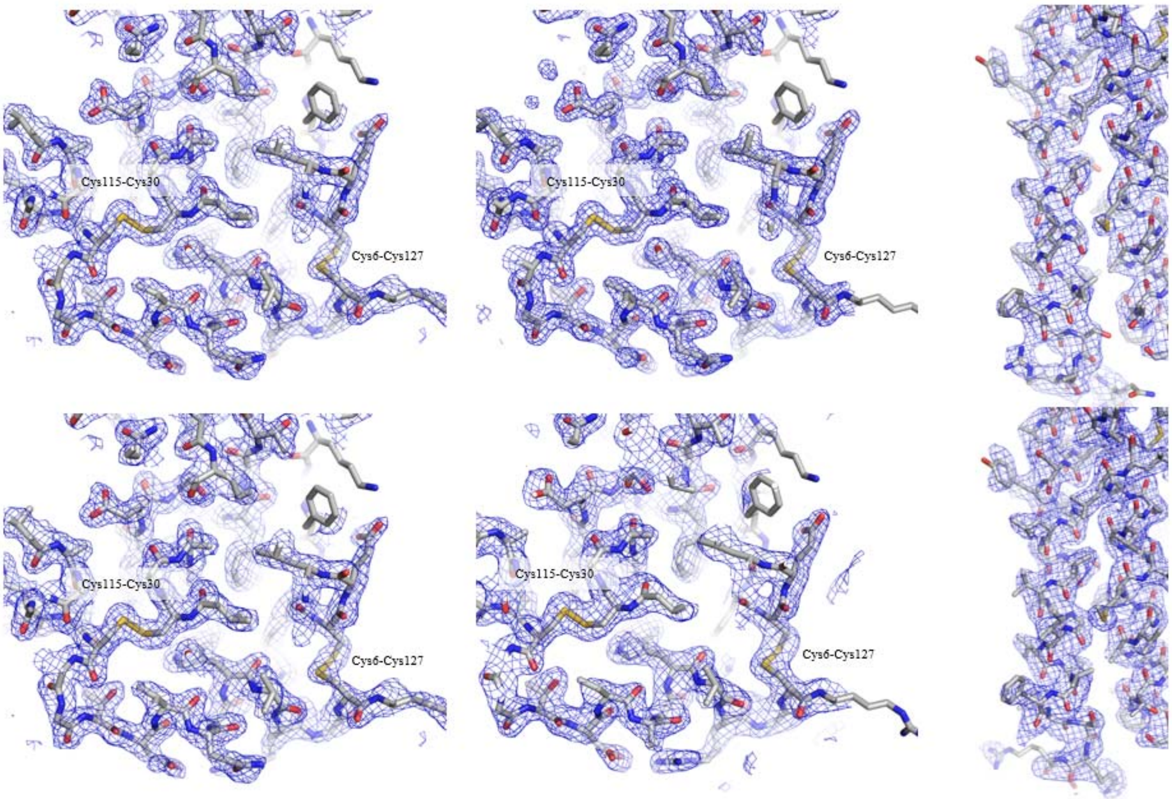

(d) (e) (f)

Figure S6 Electron-density maps for data measuring with rotation method (*a*, *b*, *c*) and still image (*d*, *e*, *f*) for lysozyme (*a*, *b, d, e*) and PepT$_{St}$ (*c*, *f*). A view for disulfide bond of Cys115-Cys30 and Cys6-Cys127 are shown for lysozyme and residues Ser352 to Ala372 and Val451 to Ile475 are shown for the PepT$_{St}$. Blue meshes is $2F_o - F_c$ maps which contoured at 1σ. (a, d, c, and f) samples were prepared using COC film. (b and e) samples were prepared using SiN chip.

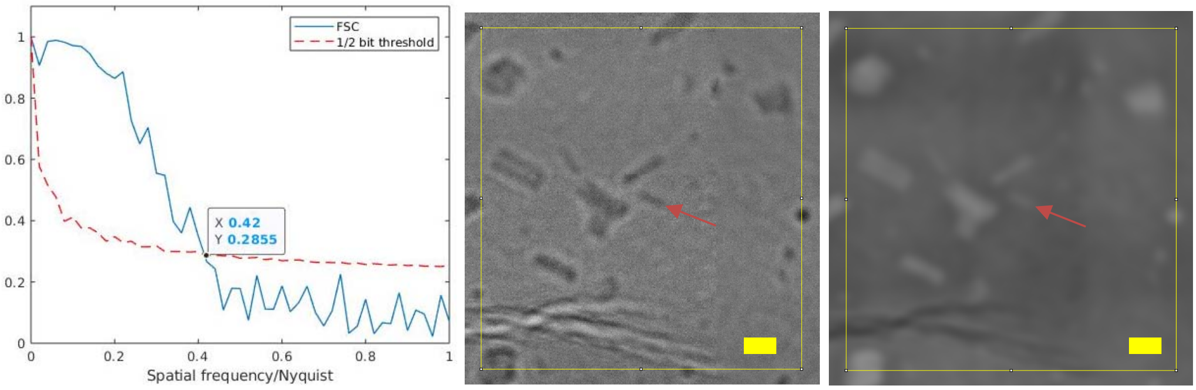

(a) (b) (c)

Figure S7 (a) Fourier ring correlation (FRC) analysis of two overlapping areas between adjacent images of the imaging grid. The FRC was performed over 100x100 pixels areas, with a half-bit threshold criterion (van Heel & Schatz, 2005). This yields a resolution of 4.76 pixels, i.e. 1.54 µm. FRC was performed before the phase retrieval step, which acts as a low-pass filter, thus worsening resolution. (b) and (c) show how an about 3-µm thick crystal (red arrow) visible before phase retrieval (b) is still visible after phase retrieval (c), showing that in this case the low-pass effect does not prevent detection of features of this size. Scale bars are 20 µm. The yellow line delimits corresponding regions.
